# RORγt may Influence the Microenvironment of Thyroid Cancer Predicting Favorable Prognosis

**DOI:** 10.1038/s41598-020-60280-3

**Published:** 2020-03-05

**Authors:** Lucas Leite Cunha, Elaine Cristina Morari, Suely Nonogaki, Natassia Elena Bufalo, Ligia Vera Montalli da Assumpção, Fernando Augusto Soares, José Vassallo, Laura Sterian Ward

**Affiliations:** 1 Laboratory of Cancer Molecular Genetics, Faculty of Medical Sciences, University of Campinas (Unicamp), Campinas, Brazil; 2 Department of Biological and Health Sciences, State University of Roraima, Boa Vista, Brazil; 3 Adolfo Lutz Institute, São Paulo, Brazil; 4 Department of General Pathology, Dental School, University of São Paulo, São Paulo, Brazil; 5 Pathology Division, ID’Or Research Institute, Rede D’Or Hospitals Network, São Paulo, Brazil; 6 Laboratory of Investigative and Molecular Pathology (Ciped), Faculty of Medical Sciences - University of Campinas (Unicamp), Campinas, Brazil

**Keywords:** Thyroid cancer, Tumour immunology

## Abstract

We aimed to investigate the role of RORγt (Retinoic acid-related orphan receptor gamma) in the tumor microenvironment of differentiated thyroid carcinoma. We retrospectively analyzed 56 patients (48 papillary and 8 follicular thyroid carcinomas). Immunohistochemical expression of RORγt was compared to other immune markers previously investigated by our group, clinical and pathological information. All patients presented cytoplasmic expression of RORγt in thyroid tumor cells. Seven (12.5%) patients presented no nuclear expression of RORγt. Positivity was few (up to 10%) in 14 patients; 10 to 50% in 5 patients (8.9%); and more than 50% in 30 patients (53.6%). Nuclear RORγt positivity was associated with absence of distant metastasis at diagnosis (p = 0.013) and the need of less cumulative doses of radioactive iodine (p = 0.039). Patients whose tumors were positive for nuclear RORγt presented higher 10-years relapse-free survival rate than those patients who were negative for RORγt (p = 0.023). We classified the patients according to the clustering of immunological immunohistochemical markers. We were able to distinguish a subset (A) of 38 patients who presented high expression of nuclear RORγt and tended to be scarce in proinflammatory immune markers. Other 16 patients integrated a second subset (B) whose tumor microenvironment accumulated proinflammatory markers and presented low expression of nuclear nuclear RORγt. Distant metastasis at diagnosis were more frequent among patients from cluster B than from cluster A (p = 0.008). Our results reinforce that the expression of RORγt together with other immune markers might help predict the prognosis of patients with thyroid cancer and help individualize clinical management.

## Introduction

The American Cancer Society’s most recent estimates for thyroid cancer in 2019 are about 52,070 new cases (14,260 in men and 37,810 in women)^1^. It is the most rapidly increasing cancer in the USA, tripling in the past three decades^1^. However, mortality rate has been steady for many years, and remains very low compared with most other cancers, suggesting widespread overdiagnosis (detection of tumors that will not cause clinical illness or death)^2–4^. Current guidelines suggest that the patients with thyroid cancer should be treated according to their overall prognosis, their risk of recurrence and mortality, the treatment’s benefits and possible troubles, and the patient’s setting and values. Unfortunately, thyroid cancer will still be responsible for about 2,170 deaths (1,020 men and 1,150 women) in the USA during 2019^1^. A series of molecular markers have been actively investigated aiming to delineate patients who would benefit of a less or of a more aggressive management, and some of them are proven to be clinically useful and are increasingly used as adjunct diagnostic molecular tests with thyroid biopsy^5^. Unfortunately, we still lack affordable, easy to perform tests capable to distinguish the low risk or indolent thyroid tumor from the one that will evolve and eventually kill the patient.

Inflammation is currently a hallmark of cancer and is known as an essential component of malignancies^6^. In fact, the tumor microenvironment is enriched with activated immune cells, which secrete cytokines and chemokines that trigger the expansion of both tumor and stromal cells^7,8^. Experimental studies have demonstrated that thyroid cancer cells express chemokine receptors, suggesting that local secretion of immune molecules may negatively influence the tumor behavior through proangiogenetic, cytoproliferative, and pro-metastatic effects^9,10^. On the other hand, the proliferation and activation of immune cells may stimulate the recognition of tumor specific antigens expressed by cancer cells leading to the tumor elimination (immunosurveillance)^7^. Tumor microenvironment inflammatory profile is closely related to the biological behavior of many tumors and our group has contributed to the knowledge in this field demonstrating that some cytokines may be associated to thyroid tumors features of aggressiveness and patients’ outcome^11,12^.

The expression of retinoic acid-related orphan receptors have been implicated in a series of diseases, with both positive and negative impact depending on conditions that include a series of factors such as autoimmunity, inflammation, metabolic syndrome, neurological disorders, and cancer. RORγt (RAR-related orphan receptor gamma T) is a transcription factor considered essential for lymphoid organogenesis^13^. RORγt regulates the thymopoiesis by reducing apoptosis of thymocytes and inducing thymocyte differentiation into pro-inflammatory T helper 17 (Th17) cells^14,15^. In tumor biology, RORγt has been associated to the promotion of lymphatic dissemination in breast cancer^16^. A recent investigation suggested that the expression of RORγt in the tumor microenvironment of papillary thyroid cancer (PTC) patients could inhibit lymph node metastasis^17^. This finding may be of great clinical importance for the best management of PTC patients. In addition, a series of studies have proposed that antagonist of the RORγ receptor has therapeutic proprieties in inflammatory diseases^18,19^, and a number of synthetic RORγ receptor antagonists have been developed^20^. RORγ agonists (such as LYC-55716) may allow the immune system to combat cancer and some compounds are in clinical trials on patients with solid tumors^21,22^.

This study aimed to better understand the role of RORγt in thyroid tumors microenvironment and its potential clinical utility.

## Material and Methods

### Patients

We retrospectively investigated consecutive patients with thyroid cancer who attended to our Thyroid Unit. Consent was obtained from each patient after full explanation of the purpose and nature of all procedures used. The Research Ethics Committee of our Hospital (A.C. Camargo Cancer Center, São Paulo, Brazil) approved our investigation. All research was performed in accordance with relevant guidelines/regulations. Written informed consent was obtained from all participants and/or their legal guardians. We excluded those patients with no histopathological confirmation of thyroid carcinoma, limited or no clinical information on their charts. We included 56 patients: 48 with papillary thyroid carcinoma (18 classic PTC, 15 follicular variant of PTC, 8 tall cells PTC and 7 poorly differentiated PTC) and 8 with follicular thyroid carcinoma (FTC). Fifty-two patients (44 with PTC and 8 with FTC) had sufficient and satisfactory tissue sample in our biobank available for molecular scrutiny of all immune markers.

Formalin-fixed paraffin-embedded tissues from all patients were reviewed for investigation of concurrent chronic lymphocytic thyroiditis (CLT) in nonmalignant thyroid parenchyma of the tumor contralateral lobe. Concurrent thyroiditis was histologically diagnosed by lymphocytic infiltration with lymphoid follicles and follicular regenerative activity with several small follicles, lined by Hurthle cells and fibrotic tissue^23^.

We classified as poorly differentiated PTC tumors with solid, trabecular or insular growth patterns, with no nuclear features of PTC and with one of the following features: convoluted nuclei, 3 or more mitotic figures per 10 high power field, and tumor necrosis^24–26^.

We evaluated presence/absence of both microscopic and gross extrathyroidal invasion. Microscopically, we considered capsular invasion and angio-lymphatic invasion. In order to stage patients, extrathyroidal extension was defined according to AJCC 8^th^ edition. Tumors with extrathyroidal extension were all of those in which gross extrathyroidal extension invaded only strap muscles from a tumor of any size.

Among the follicular variant of PTC cases, there were 4 patients presenting the infiltrative subtype and 11 patients presenting the encapsulated subtype. Among the FTC cases, we included 2 patients with minimally invasive FTC and 6 patients with widely invasive FTC. Five patients presented a unifocal lesion, while multifocality was found in 3/8 patients. Gross extrathyroid extension was observed in one patient; microscopically vascular invasion was observed in 6/8 patients. None of the patients with FTC presented lymph-node metastasis at diagnosis. Distant metastasis was observed in 3/8 patients. Three patients presented oxyphilic cells in tumor parenchyma and one patient presented trabecular/insular growth pattern.

We used pTNM classification system (AJCC Cancer Staging Manual 8^th^ edition) for thyroid cancer as criteria of aggressiveness at diagnosis^27^. Patients were followed-up by standard protocol that include periodic total body scans, serum thyroglobulin and thyroid-stimulating hormone measurements, radiologic scrutiny (X-ray, ultrasonography, computed tomography scan) and other approaches to detect local or distant metastasis for a period of 16–322 months (157.7 ± 68.3 months). During the follow-up, patients presenting elevated non-stimulated serum thyroglobulin levels (>2 mg/dl) were submitted to a meticulous image search. The mentioned clinical parameters were used to define tumors as persistent/recurrent and/or presenting long distance metastasis. Patients were considered free-of disease when they evolved with stable unstimulated serum thyroglobulin levels <2 ng/dl or undetectable thyroglobulin levels for more than 2 years after tumor resection, without any suspicion of recurrence^28^. We considered 42 patients free-of-disease and 14 presented recurrence/metastasis during the observation period. Two patients from our cohort died due to thyroid cancer during follow-up.

### Immunohistochemistry and semiquantification

Samples from all specimens were reviewed with the intention to sort the most representative area designed to build a tissue microarray (Beecher Instruments®, Silver Springs, MD, USA) for immunohistochemical analysis. Then, we obtained 4 tumor tissue cores from each patient: 2 spots were selected from representative area enriched with leukocytes and 2 other spots from representative area free of leukocytes. The use of two spots has been widely used in pathology and has shown good correlation to whole sections analyses.

**Figure 1:**
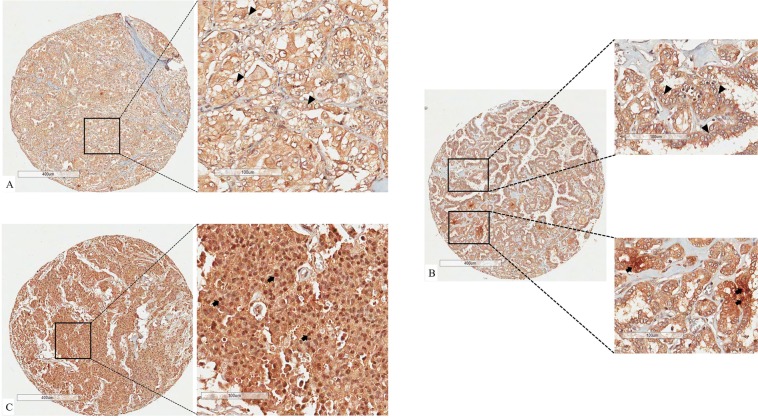
Different percentages of nuclear RORγt positivity. Panel (A) shows the diffuse brownish in cytoplasm but not in nuclear of thyroid carcinoma cells. Panel (B) evidence that major of nuclei of thyroid cancer cells are negative for RORγt expression (upper right panel). Lower right panel shows a detail of focus of nuclei positive for RORγt. Panel C represent a tissue spot in which all nuclei of thyroid cancer cells are positive for RORγt. Black arrows point to nuclei positive for RORγt. Black arrow head point to nucleai negative for RORγt. Stromal components like collagen, vessels and myocytes are also not expected to express nuclear RORγt, giving us a negative control for each spot analyzed.

We investigated the expression of IL-1β, IL-17, IL-23, IL-10 and COX2 in tumor cells. We looked for intratumoral infiltration of immune cells using CD3, CD4, CD8, CD20, CD68, CD16 and CD45RO markers. Activated immune cells were analyzed using CD25, CD69 and Granzyme B markers. Additionally, we studied the expression of RORγt in tumors cells (Fig. 1). Immunohistochemistry was performed as previously described, using positive and negative controls in the same batch of reactions^29^. We used human reactive lymph node as positive control. Negative control was obtained performing the same batch of reaction in the same reactive lymph node, but, without adding primary antibody.

Semiquantification was performed by two investigators (LLC and ECM), as routinely done by our group^30–32^. Two experienced pathologists (JV and FAS) blinded to tumor information evaluated all slides. Cells were defined as positive for immunohistochemical markers when a clear cut brown staining was observed in the corresponding cellular localization. Visual analysis of aforementioned markers was done considering an approximate area of 0.79 mm^2^ per tissue microarray spot. We estimated the markers of tumor cells by the percentage of positive tumor cells and the intensity of its staining. We assessed the infiltration of immune cells classifying them as 0 (no immune cells infiltration), 1 (infiltration of up to 10 immune cells) and 2 (infiltration of more than 10 immune cells in each spot). RORγt was cytoplasmic expressed in tumor cells from virtually all samples. Then, we categorized the patients according to the percentage of tumor cells harboring nuclear expression of RORγt. Patients were classified as 0 (no tumor cells with nuclear expression of RORγt), 1 (1 to 10% of tumor cells positive for nuclear expression of RORγt), 2 (11 to 50% of tumor cells positive for nuclear expression of RORγt) and 3 (more than 50% of tumor cells positive for nuclear expression of RORγt).

### Cluster and statistical analysis

We performed the hierarchical cluster analysis of our immunohistochemical data. Cluster 3.0 was used to assess similarities/distances of patients according to the assembled immunological results. Following the instruction of the authors, the eight parameters (RORγt, CD8, CD16, COX2, IL-1β, IL-17A, IL-10 and IL-23) were submitted to complete-linkage hierarchical clustering. Java TreeView was used to graphically construct the dendrogram that express similarities/distance of patients.

Statistical analysis was performed using the Statistical Package for the Social Sciences (SPSS) ® software, version 23.0. First relapse was defined as end-point. Kaplan-Meier method was used to calculate relapse-free survival rates and survival were compared using log-rank test. Chi-square or Fisher’s exact test were assessed to perform nonparametric analysis, when indicated. Mann-Whitney tests were used to compare continuous measures between two independent groups whose variables did not fit to normal distribution; Kruskal–Wallis test was performed to compare continuous measures between three or more groups whose variables did not fit normal distribution. All tests were conducted at a 0.05 significance level.

### Compliance with ethical standards

Informed consent was obtained from each patient after full explanation of the purpose and nature of all procedures used. The Research Ethics Committee of our Hospital (A.C. Camargo Cancer Center, São Paulo, Brazil) approved our investigation

## Results

Our cohort included 46 female and 10 male patients. Mean age at diagnosis was 42 ± 17.3 years; mean tumor size was 3.1 ± 2.2 cm. Twenty-eight patients (50%) presented multifocality. Eleven patients (20%) presented capsular invasion, 13 (23.6%) presented angio-lymphatic invasion, 14 (25.5%) presented extrathyroid invasion and 30 (54.4%) presented capsular invasion, vascular invasion or gross extrathyroidal invasion at the diagnosis. Fourteen (25%) patients had evidence of concurrent thyroiditis. Twenty-three (41.8%) patients presented lymph-node involvement at diagnosis. Nine (16.1%) patients presented long distant metastasis at diagnosis. The majority of patients were diagnosed in early stage of the disease (pTNM I; n = 28; 50%), whereas 13 (23.2%) patients were diagnosed in pTNM II; 7 (12.5%) patients were diagnosed in pTNM III; and 8 (14.3%) patients were diagnosed in pTNM IV. Mean cumulative dose of ^131^I-iodine therapy was 269 mCi (±260 mCi, ranging from 0 to 1200 mCi). Table 1 summarizes the correlation between clinic and pathological features of patients with thyroid cancer and different tumor sub-types.

**Table 1:** Correlation between clinic and pathological features of patients with thyroid cancer and different tumor sub-types.

	Classic PTC	FV-PTC	TC-PTC	PD-PTC	FTC	p-value*	p-value**	p-value***
**Gender**								
Female	14 (77.8)	12 (80.0)	7 (87.5)	7 (100.0)	6 (75.0)	0.692	0.568	0.569
Male	4 (22.2)	3 (20.0)	1 (12.5)	0 (0.0)	2 (25.0)			
Age, Mean ± SD	33.7 ± 11.8	43.1 ± 17.3	51.4 ± 15.2	38.3 ± 19.7	58.5 ± 16.3	**0.006**	0.056	**0.022**
**Multifocality**								
Unifocal	9 (50.0)	7 (46.7)	4 (50.0)	3 (42.9)	5 (62.5)	0.950	0.988	0.445
Multifocal	9 (50.0)	8 (53.3)	4 (50.0)	4 (57.1)	3 (37.5)			
**Concurrent CLT**								
Absent	13 (72.2)	11 (73.3)	6 (75.0)	6 (85.7)	6 (75.0)	0.971	0.913	1.000
Present	5 (27.8)	4 (26.7)	2 (25.0)	1 (14.3)	2 (25.0)			
**pTNM**								
I	10 (55.6)	9 (60.0)	2 (25.0)	6 (85.7)	1 (12.5)	**0.035**	**0.025**	0.073
II	4 (22.2)	4 (26.7)	1 (12.5)	1 (14.3)	3 (37.5)			
III	3 (16.7)	2 (13.3)	1 (12.5)	0 (0.0)	1 (12.5)			
IV	1 (5.6)	0 (0.0)	4 (50.0)	0 (0.0)	3 (37.5)			
**Lymph-node metastasis at diagnosis**								
Absent	7 (38.9)	10 (66.7)	3 (37.5)	5 (71.4)	8 (100.0)	**0.028**	0.234	**0.011**
Present	11 (61.1)	5 (33.3)	5 (62.5)	2 (28.6)	0 (0.0)			
**Distant metastasis at diagnosis**								
Absent	16 (88.9)	15 (100.0)	6 (75.5)	6 (87.5)	5 (62.5)	0.140	0.297	**0.043**
Present	2 (11.1)	0 (0.0)	2 (25.5)	1 (14.3)	3 (37.5)			
**Capsular invasion**								
Absent	17 (94.4)	11 (73.3)	7 (87.5)	6 (85.7)	4 (50.0)	0.114	0.429	**0.022**
Present	1 (5.6)	4 (26.7)	1 (12.5)	1 (14.3)	4 (50.0)			
**Vascular invasion**								
Absent	15 (83.3)	13 (86.7)	5 (62.5)	7 (100.0)	3 (37.5)	**0.027**	0.266	**0.005**
Present	3 (16.7)	2 (13.3)	3 (37.5)	0 (0.0)	5 (62.5)			
**Gross extrathyroid extension**								
Absent	14 (77.8)	15 (100.0)	1 (12.5)	5 (71.4)	7 (87.5)	**<0.001**	**<0.001**	0.363
Present	4 (22.2)	0 (0.0)	7 (87.5)	2 (28.6)	1 (12.5)			
**Any invasion**								
Absent	12 (66.7)	10 (66.7)	1 (12.5)	3 (42.9)	0 (0.0)	**0.004**	0.055	**0.005**
Present	6 (33.3)	5 (33.3)	7 (87.5)	4 (57.1)	8 (100.0)			
RAI doses, Mean ± SD	220.4 ± 173.8	268.0 ± 302.1	406.2 ± 322.3	142.8 ± 142.7	356.2 ± 323.4	0.201	0.123	0.548

Abbreviations: SD, standard deviation; CLT, chronic lymphocytic thyroiditis; PTC, papillary thyroid carcinoma; FV-PTC, follicular variant of PTC; TC-PTC, tall cell variant of PTC; PD-PTC, poorly differentiated, PTC; FTC, follicular thyroid carcinoma; RAI, radioactive iodine therapy cumulative doses.

Note: *comparison considering all different histologic subtypes (5 categories); **comparison considering different subtype of PTC (4 categories); ***comparison considering PTC versus FTC.

Among the patients with gross extrathyroidal extension, 84.6% of them also presented lymph-node metastasis at diagnosis (p = 0.001), and 23.1% of them presented distant metastasis at diagnosis (p = 0.027). Patients with PTC and gross extrathyroidal extension presented a poor prognosis compared with those with no gross extrathyroidal extension at the diagnosis (46,2% of five-years survival rate versus 88%, respectively; p-value= 0.001), reinforcing that gross extrathyroidal extension is associated with PTC aggressiveness.

**Figure 2:**
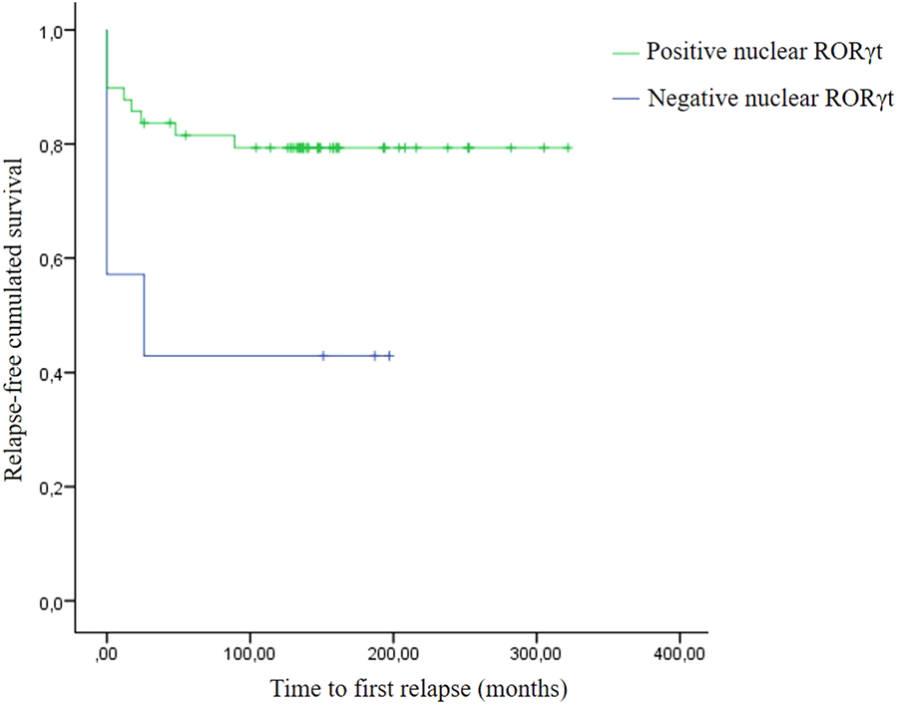
Kaplan-Meier curve shows that patients whose tumors were positive for RORγt presented favorable outcome with higher relapse-free survival rate.

All patients presented cytoplasmic expression of RORγt in thyroid tumor cells. Since cytoplasm is not the natural subcellular location of RORγt, we investigated its nuclear expression by semiquantitative estimation of nuclear RORγt positivity in tumor cells, as demonstrated in Fig. 1. Seven (12.5%) patients presented no nuclear expression of RORγt; 14 patients (25.0%) presented few (1 to 10%) nuclear positivity; 5 patients (8.9%) presented 11 to 50% of positivity; and 30 patients (53.6%) presented more than 50% of positivity. Table 2 shows the association between nuclear expression of RORγt and clinical features of the patients. We found an association between RORγt positivity and absence of distant metastasis at diagnosis (p = 0.013). Patients whose tumors were positive for nuclear RORγt required less cumulative doses of radioactive iodine (RAI; p = 0.039). We did not observe any difference neither concerning relapse-free survival nor in overall survival regarding RORγt positivity. In order to accurately assess the prognostic impact of RORγt, we gathered patients with 1–10% of nuclear positivity (14 patients), patients with 11–50% of nuclear positivity (5 patients) and patients who presented more than 50% of nuclear positivity (30 patients) which were compared with patients whose tumors presented no nuclear positivity for RORγt. Patients whose tumors were positive for nuclear RORγt presented higher 10-years relapse-free survival rate (79.3%) than those patients who were negative for RORγt (42.9%; p = 0.023; Fig. 2).

**Table 2:** Comparison of RORγt positivity and clinical and pathological features of aggressiveness of differentiated thyroid carcinomas.

Clinical feature	Estimation of percentage of tumor cells expressing nuclear RORγt				p-value^a^
	0%	1–10%	11–50%	> 50%	
**Gender**					
Male	1 (10.0)	2 (20.0)	1 (10.0)	6 (60.0)	0.961
Female	6 (13.0)	12 (26.1)	4 (8.7)	24 (52.2)	
**Age at diagnosis**					
45 yrs or less	3 (11.1)	9 (33.3)	2 (7.4)	13 (48.1)	0.583
More 45 yrs	4 (13.8)	5 (17.2)	3 (10.3)	17 (58.6)	
**Histologic types**					
Classic PTC	2 (11.1)	8 (44.4)	0 (0.0)	8 (44.4)	0.381*
FV-PTC	1 (6.7)	4 (26.7)	2 (13.3)	8 (53.3)	0.280**
TC-PTC	1 (12.5)	0 (0.0)	2 (25.0)	5 (62.5)	0.592***
PD-PTC	1 (14.3)	1 (14.3)	0 (0.0)	5 (71.4)	
FTC	2 (25.0)	1 (12.5)	1 (12.5)	4 (50.0)	
**Multifocality**					
Unifocal	2 (7.1)	7 (25.0)	4 (14.3)	15 (53.6)	0.379
Multifocal	5 (17.9)	7 (25.0)	1 (3.6)	15 (53.6)	
**Concurrent CLT**					
Absent	7 (16.7)	10 (23.8)	4 (9.5)	21 (50.0)	0.408
Present	0 (0.0)	4 (28.6)	1 (7.1)	9 (64.3)	
Tumor size, Mean ± SD	4.6 ± 2.9	2.8 ± 1.5	3.7 ± 1.9	2.8 ± 2.4	0.185
**LN metastasis at diagnosis**					
Absent	3 (9.1)	6 (18.2)	2 (6.1)	22 (66.7)	0.163
Present	4 (17.4)	8 (34.8)	3 (13.0)	8 (34.8)	
**Distant metastasis at diagnosis**					
Absent	3 (6.4)	12 (25.5)	4 (8.5)	28 (59.6)	**0.013**
Present	4 (44.5)	2 (22.2)	1 (11.1)	2 (22.2)	
RAI doses, Mean ± SD	492.8 ± 386.7	247.6 ± 173.1	254.0 ± 127.8	230.0 ± 261.5	**0.039**

Abbreviations: SD, standard deviation; CLT, chronic lymphocytic thyroiditis; PTC, papillary thyroid carcinoma; FV-PTC, follicular variant of PTC; TC-PTC, tall cell variant of PTC; PD-PTC, poorly differentiated, PTC; FTC, follicular thyroid carcinoma; RAI, radioactive iodine therapy cumulative doses.

Note: ^a^The p-value expressed was obtained when the comparison was done considering the four different categories separated and not combined. *Comparison considering all different histologic subtypes (5 categories); **comparison considering different subtype of PTC (4 categories); ***comparison considering PTC versus FTC.

We observed IL-17A expression in cytoplasm of thyroid cancer cells from all patients. Interestingly, 42 patients (75.0%) presented a faint cytoplasmic expression of IL-17A, while strong diffuse expression was observed in the remaining 14 patients (25.0%). We, then, investigated association between clinical data and IL-17A, considering faint versus strong IL-17A expression. IL-17A failed to be associated with clinical and pathological characteristics of aggressiveness.

Thirty-two patients (58.1%) presented expression of IL-1β, all of them in cytoplasmic subcellular location; twenty-three patients (41.8%) presented no expression of IL-1β. One patient presented a doubtful expression and was excluded from further analysis. Table 3 summarizes associations between IL-1β and clinical and pathological features of patients. IL-1β expression was observed more frequently among patients who presented no evidence of concurrent CLT (p = 0.048), with distant metastasis at diagnosis (p = 0.041). In addition, patients whose tumors presented IL-1β expression were diagnosed with larger tumor size (p = 0.032) and required a higher RAI cumulative doses (p = 0.004). Patients whose tumors were positive for IL-1β tended to present lower 10-years relapse-free survival rate (65.3%) than patients who were negative for IL-1β (87.0%), but this difference was marginally significant (p = 0.081).

**Table 3:** Comparison of IL-1β positivity and clinical and pathological features of aggressiveness of differentiated thyroid carcinomas.

Clinical feature	Estimation of positivity for IL-1β		p-value
	Negative	Positive	
**Gender**			
Male	4 (40.0)	6 (60.0)	0.897
Female	19 (42.2)	26 (57.8)	
**Age at diagnosis**			
45 yrs or less	11 (42.3)	15 (57.7)	0.944
more 45 yrs	12 (41.4)	17 (58.6)	
**Histologic types**			
Classic PTC	9 (50.0)	9 (50.0)	0.278*
FV-PTC	5 (33.3)	10 (66.7)	0.268**
TC-PTC	2 (25.0)	6 (75.0)	0.297***
PD-PTC	5 (71.4)	2 (28.6)	
FTC	2 (25.0)	6 (75.0)	
**Multifocality**			
Unifocal	11 (39.3)	17 (60.7)	0.698
Multifocal	12 (44.4)	15 (55.6)	
**Concurrent CLT**			
Absent	14 (34.1)	27 (65.9)	**0.048**
Present	9 (64.3)	5 (35.7%)	
Tumor size, Mean ± SD	2.4 ± 2.0	3.7 ± 2.3	**0.032**
**LN metastasis at diagnosis**			
Absent	15 (45.5)	18 (54.5)	0.503
Present	8 (36.4)	14 (63.6)	
**Distant metastasis at diagnosis**			
Absent	22 (46.8)	25 (53.2)	**0.041**
Present	1 (12.5)	7 (87.5)	
RAI doses, Mean ± SD	178.2 ± 206.0	332.4 ± 282.8	**0.004**

Abbreviations: SD, standard deviation; CLT, chronic lymphocytic thyroiditis; PTC, papillary thyroid carcinoma; FV-PTC, follicular variant of PTC; TC-PTC, tall cell variant of PTC; PD-PTC, poorly differentiated, PTC; FTC, follicular thyroid carcinoma; RAI, radioactive iodine therapy cumulative doses.

Note: *Comparison considering all different histologic subtypes (5 categories); **comparison considering different subtype of PTC (4 categories); ***comparison considering PTC versus FTC.

Thirty-nine patients (69.6%) presented cytoplasmic homogeneous expression of IL-23; the remaining 17 patients presented no expression of IL-23. As seen in Table 4, expression of IL-23 was more frequently found in patients who presented distant metastasis at diagnosis (p = 0.031). Patients whose tumors presented IL-23 expression were diagnosed with larger tumor size (p = 0.009) and required a higher RAI cumulative doses (p = 0.037). Log-rank test did not evidence the expression of IL-23 as predictor of relapse-free survival (data not shown).

**Table 4:** Comparison of IL-23 positivity and clinical and pathological features of aggressiveness of differentiated thyroid carcinomas.

Clinical Feature	Estimation of positivity for IL-23		
	Negative	Positive	p-value
**Gender**			
Male	3 (30.0)	7 (70.0)	0.978
Female	14 (30.4)	32 (69.6)	
**Age at diagnosis**			
45 yrs or less	9 (33.3)	18 (66.7)	0.640
more 45 yrs	8 (27.6)	21 (72.4)	
**Histologic types**			
Classic PTC	8 (44.4)	10 (55.6)	0.019*
FV-PTC	2 (13.3)	13 (86.7)	0.020**
TC-PTC	1 (12.5)	7 (87.5)	0.235***
PD-PTC	5 (71.4)	2 (28.6)	
FTC	1 (12.5)	7 (87.5)	
**Multifocality**			
Unifocal	7 (25.0)	21 (75.0)	0.383
Multifocal	10 (35.7)	18 (64.3)	
**Concurrent CLT**			
Absent	11 (26.2)	31 (73.8)	0.240
Present	6 (42.9)	8 (57.1)	
Tumor size, Mean ± SD	2.2 ± 2.2	3.6 ± 2.2	**0.009**
**LN metastasis at diagnosis**			
Absent	10 (30.3)	23 (69.7)	0.949
Present	7 (30.4)	16 (69.6)	
**Distant metastasis at diagnosis**			
Absent	17 (36.2)	30 (63.8)	**0.031**
Present	0 (0.0)	9 (100.0)	
RAI doses, Mean ± SD	158.8 ± 153.3	317.6 ± 283.9	**0.037**

Abbreviations: SD, standard deviation; CLT, chronic lymphocytic thyroiditis; PTC, papillary thyroid carcinoma; FV-PTC, follicular variant of PTC; TC-PTC, tall cell variant of PTC; PD-PTC, poorly differentiated, PTC; FTC, follicular thyroid carcinoma; RAI, radioactive iodine therapy cumulative doses.

Note: *comparison considering all different histologic subtypes (5 categories); **comparison considering different subtype of PTC (4 categories); ***comparison considering PTC versus FTC.

**Figure 3:**
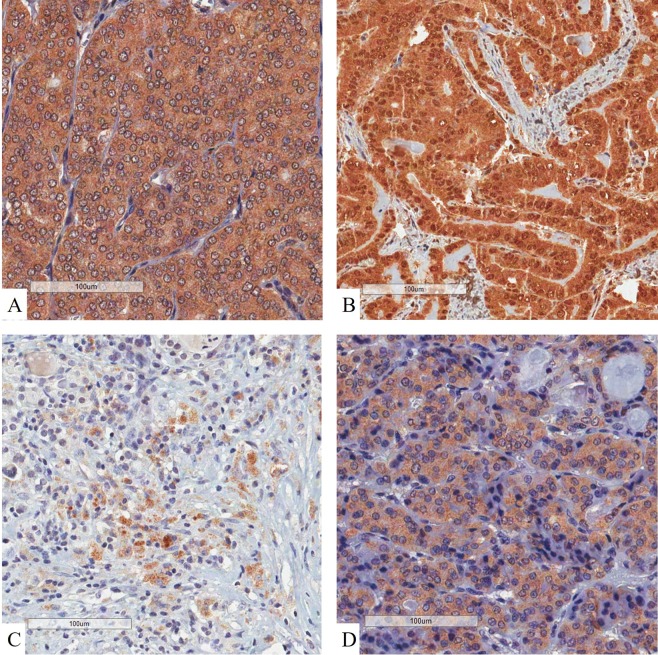
Immunohistochemistry of interleukins. (**A**) IL-1β presented a diffuse, homogeneous and cytoplasmic pattern of expression in thyroid cancer cells. (**B**) IL-10 was seen as strong brownish expression in both nuclei and cytoplasm of malignant cells. (**C**) IL-17A presented a focal cytoplasmic expression among thyroid cancer cells. (**D**) IL-23 expression was observed in cytoplasm of malignant cells. All panels evidenced that stromal structures (eg. collagen, colloid and vessels) lack expression of immune markers.

We compared nuclear RORγt, IL-17A, IL-1β and IL23 expression with other immune markers previously investigated by our group^31^ (Table 5). Nuclear RORγt was inversely associated with infiltration of CD16 + lymphocytes (p = 0.047). Likewise, nuclear RORγt expression was inversely correlated to COX2 (p = 0.024), IL-1β (p = 0.001) and IL23 (p = 0.043). IL-1β was directly associated with infiltration of CD8 + lymphocytes (p = 0.002), infiltration of CD16 + lymphocytes (p = 0.046), expression of COX2 (p < 0.001), IL-10 (p = 0.032) and IL23 (p < 0.001). IL-23 was directly associated with infiltration of CD8 + lymphocytes (p = 0.004), infiltration of CD16 + lymphocytes (p = 0.003) and expression of COX2 (p < 0.001). Figure 3 shows the immunostaining for the investigated interleukins.

**Table 5:** Association between immune markers to each other.

Immune markers	Immune markers			
	RORγt	IL-17A	IL-1β	IL-23
CD4	0.823	0.152	0.458	0.743
CD8	0.452	0.320	0.002^d^	0.004^d^
CD20	0.207	0.593	0.098	0.978
CD68	0.537	0.750	0.924	0.264
CD16	0.047^i^	0.186	0.046^d^	0.003^d^
CD25	0.537	0.453	0.421	0.239
CD69	0.524	0.416	0.306	0.084
Granzyme B	0.061	0.914	0.125	0.188
CD45RO	0.521	0.759	0.795	0.354
CD134	0.380	0.702	0.358	0.110
COX2	0.024^i^	0.169	<0.001^d^	<0.001^d^
IL-10	0.162	0.275	0.032^d^	0.141
IL-23	0.043^i^	0.308	<0.001^d^	
IL-1β	0.001^i^	0.163		
IL-17A	0.035^i^			

Abbreviations: d, direct association; i, inverse association. Further molecular association between IL-10, Cox2, CD3, CD4, CD8, CD20, CD68, CD16, CD45RO, CD25, CD69 and Granzyme B was previously published by our group^28,31,44^.

**Figure 4:**
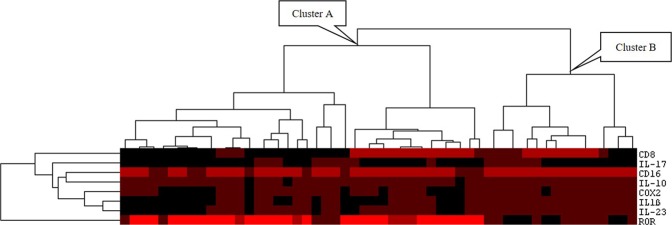
Dendrogram shows hierarchical cluster that classified patients in two different group. Group A represents patients who presented high expression of nuclear RORγt and tended to be scarce in proinflammatory immune markers. Group B represents patients whose tumor microenvironment accumulated proinflammatory markers and presented low expression of nuclear nuclear RORγt.

After accessing the association of immunological markers with each other, we classified the patients according to the clustering of the significant markers (RORγt, CD8, CD16, COX2, IL-1β, IL-17A, IL-10 and IL-23). The complete immunohistochemical characterization of the eight variables was available for 54 patients. Figure 4 shows the dendrogram obtained after cluster immunological markers. Thirty-eight patients were categorized in the first subset of subjects (cluster A) and most of them presented nuclear expression of RORγt. Sixteen patients were categorized in the second subset of subjects (cluster B). Different tumor types were not associated to molecular cluster (Table 6). Distant metastasis at diagnosis was more frequently found among patients from cluster B (37.5%) than among patients from cluster A (7.9%; p = 0.008). Patients from cluster A presented higher 10-years relapse-free survival rate (78.5%) than those patients from cluster B (62.5%; p = 0.185), but the difference was not significant.

**Table 6:** Association between cluster of molecular markers and different tumor subtypes.

Histologic types	Cluster of molecular markers		
	A	B	p-value
Classic PTC	11 (73.3)	4 (26.7)	0.540*
FV-PTC	11 (78.6)	3 (21.4)	0.859**
TC-PTC	7 (87.5)	1 (12.5)	0.134***
PD-PTC	5 (71.4)	2 (28.6)	
FTC	8 (100.0)	0 (0.0)	

Abbreviations: PTC, papillary thyroid carcinoma; FV-PTC, follicular variant of PTC; TC-PTC, tall cell variant of PTC; PD-PTC, poorly differentiated, PTC; FTC, follicular thyroid carcinoma.

Note: *comparison considering all different histologic subtypes (5 categories); **comparison considering different subtype of PTC (4 categories); ***comparison considering PTC versus FTC.

## Discussion

Our results showed that nuclear RORγt expression was associated to absence of distant metastasis at diagnosis and lower requirement of therapeutic RAI dose. Besides identifying less aggressive tumors, nuclear RORγt expression might predict favorable outcome in patients with thyroid carcinoma. Nuclear RORγt was negatively correlated to expression of IL-17A, IL-1β and IL-23, all cytokines engaged in promotion of Th17 related immune response. Interestingly, on the contrary of nuclear RORγt expression, both IL-1β and IL-23 were correlated to more aggressive clinic and pathological features that included large tumor size, presence of distant metastasis at diagnosis and requirement of higher cumulative dose of RAI. In fact, when we gather the molecular markers through hierarchical cluster, patients whose tumor microenvironment was enriched with pro-inflammatory Th17-related cytokines frequently presented distant metastasis at diagnosis, suggesting that IL-1β and IL-23 may help to promote tumor invasion, while nuclear RORγt might inhibit tumor dissemination.

Zeng *et al*. obtained similar results^17^. Nuclear RORγt expression was inversely correlated to lymph node metastasis both in patients with PTC alone and in patients with concurrent Hashimoto’s thyroiditis, corroborating our findings and suggesting that RORγt might have an anti-tumor role in the tumor microenvironment. In fact, data from experimental investigation evidenced that RORγt agonist not only decreases the percentage of PD-1+ cells but also reduces the level of PD-1 in individual immune cells^32^, suggesting that RORγt may blunt T cell exhaustion, potentiate anti-tumor T cell response and diminish pro-tumor inflammatory response^18,32^. In addition, Hu *et al*. investigated tumor-bearing mice that received oral RORγt agonist compared to mice that received vehicle control. Mice that received oral RORγt agonist presented an inhibition of tumor growth and longer survival compared to control^32^, reinforcing the idea that RORγt may be a potential therapeutic target for patients with cancer, including thyroid carcinoma.

We failed to correlate IL-17A with any clinical and pathological predictors of prognosis. On the contrary, Zeng *et al*. showed that the peripheral serum concentration of IL-17A was positively correlated with local invasion in PTC patients with no concurrent thyroiditis. When there was Hashimoto’s thyroiditis concurrent to PTC, a negative correlation between IL-17A concentration and lymph node metastasis was observed. The reason why IL-17A accumulation behaves differently depending of the concurrence of Hashimoto´s thyroiditis remains unclear. The concurrent autoimmunity may influence tumor microenvironment and help to sabotage the pro-tumor tendency of inflammatory response^31,33^. Carvalho *et al*. found a higher percentage of cells expressing IL-17 in patients with thyroid cancer^34^. Additionally, IL-17 but not IL-23 expression was associated with recurrence/mortality, suggesting that the enrichment of IL-17A in tumor microenvironment may be related to aggressiveness in thyroid cancer^34^. It is worthy to note that both pro- and anti-tumors role have been linked to IL-17A^35,36^. These apparently conflicting data may be due to the multi-faceted immune responses associated with Th17 cells while IL-17A, as a unique cytokine, may reveal its effects depending on the tumor microenvironment and tumor histology^32,34,35^. Interestingly, Carvalho *et al*. found a positive correlation between IL-17A and IL-23 expression. Likewise, we observed a tight correlation between IL-17A, IL-23, IL-1β, all cytokines related to the Th17 arm of immune response. More studies are warranted to unveil the specific role of IL-17A in tumor milieu of differentiated thyroid carcinoma.

Also, we demonstrated that patients whose tumors were positive for IL-1β required higher RAI cumulative doses, suggesting that IL-1β may be associated to RAI resistance. RAI gets into thyroid cells by the sodium iodide symporter (NIS). Spitzweg *et al*. investigated the modulation of NIS expression and NIS activity in thyroid cell lines^37^. Incubation of thyroid cells with IL-1β resulted in a 30% decrease of NIS mRNA steady-state levels^37^. IL-1β suppressed iodide accumulation by approximately 25%^37^, favoring the idea that tumors that accumulate IL-1β may decrease NIS expression and activity, explaining why higher dose of RAI is required in those patients. Both autocrine and paracrine production of IL-1β may happen in tumor microenvironment and malignant thyroid follicular cells are capable of recognize bacterial lipopolysaccharide and elicit IL-1β production^38^. Many human cancers express IL-1β and its overproduction is associated with poor prognosis^39,40^. IL-1β polymorphisms that leads to overexpression of IL-1β might be a predictive factor for lymph node metastasis of PTC patients^41^, reinforcing the idea that IL-1β may help to promote tumor invasion and dissemination.

Our data have some limitations. The use of TMA could reduce the size of tumor samples, which may be seen as a limitation to its use. However, in a previous study on thyroid carcinoma, ROR mRNA was evaluated by *in situ* hybridization in TMA, and a good correlation was found with RT-PCR^42^. We believe that the results of the present study are representative of the real degree of ROR protein expression in PTC. We obtained the clinical and pathological information from the patient’s charts retrospectively. Further prospective studies should dismiss this putative bias. In addition, we investigated protein expression of RORγt and Th17 related cytokines. We could not accurately assess how these molecules interact to each other *in vitro* and the undoubted role of the cytokines in the tumor microenvironment remains to be elucidated. In fact, most of literature investigated the nuclear RORγt, IL-17A, IL-23 and IL-1β expression in lymphocytes, but not in epithelial cells. Herein, we reported the nuclear RORγt, IL-17A, IL-23 and IL-1β in tumor cells of papillary thyroid cancer. Therefore, it is possible that the molecular relationships between RORγt and IL-17A, IL-23 and IL-1β seen on lymphocytes cannot be extrapolated to tumor cells. In addition, we were not able to find any association between the investigated markers and patients’ outcome. In fact, current therapy for DTC is very effective and, although excessive for most patients, it certainly contributes to the excellent prognosis of the patients, impairing long-term evaluation of the role of specific factors.

In summary, our data evidence that RORγt is expressed in nuclei of PTC cells and this expression is associated with clinical and pathological features of favorable prognosis, suggesting that RORγt may favor anti-tumor immune response in the microenvironment of thyroid cancer. In fact, RORγt was previously associated to favorable prognosis in renal and colorectal cancer^43^. On the contrary, IL-23 and IL-1β are associated to distant metastasis at diagnosis suggesting these cytokines may facilitate a pro-tumor inflammatory response engaged in tumor dissemination and aggressiveness.

Immunohistochemical expression of RORγt, IL-23 and IL-1β can be easily accessed in routine pathology laboratories helping to predict the prognosis of patients with thyroid cancer and better individualize their clinical management. A personalized individual clinical approach is of utmost need in order to consider the effect of different immune markers and their relationship.
